# Revisiting tumor angiogenesis: vessel co-option, vessel remodeling, and cancer cell-derived vasculature formation

**DOI:** 10.1186/s40880-015-0070-2

**Published:** 2016-01-08

**Authors:** Chao-Nan Qian, Min-Han Tan, Jun-Ping Yang, Yun Cao

**Affiliations:** State Key Laboratory of Oncology in South China Collaborative, Innovation Center for Cancer Medicine, Sun Yat-sen University Cancer Center, Guangzhou, 510060 Guangdong P.R. China; Department of Nasopharyngeal Carcinoma, Sun Yat-sen University Cancer Center, Guangzhou, 510060 Guangdong P.R. China; Institute of Bioengineering and Nanotechnology, Singapore, Republic of Singapore; Department of Pathology, Sun Yat-sen University Cancer Center, Guangzhou, 510060 Guangdong P.R. China

**Keywords:** Angiogenesis, Vessel co-option, Vessel-like structure, Vessel remodeling, Vascular endothelial growth factor

## Abstract

Tumor growth and metastasis depend on the establishment of tumor vasculature to provide oxygen, nutrients, and other essential factors. The well-known vascular endothelial growth factor (VEGF) signaling is crucial for sprouting angiogenesis as well as recruitment of circulating progenitor endothelial cells to tumor vasculature, which has become therapeutic targets in clinical practice. However, the survival benefits gained from targeting VEGF signaling have been very limited, with the inevitable development of treatment resistance. In this article, we discuss the most recent findings and understanding on how solid tumors evade VEGF-targeted therapy, with a special focus on vessel co-option, vessel remodeling, and tumor cell-derived vasculature establishment. Vessel co-option may occur in tumors independently of sprouting angiogenesis, and sprouting angiogenesis is not always required for tumor growth. The differences between vessel-like structure and tubule-like structure formed by tumor cells are also introduced. The exploration of the underlying mechanisms of these alternative angiogenic approaches would not only widen our knowledge of tumor angiogenesis but also provide novel therapeutic targets for better controlling cancer growth and metastasis.

## Background

Normal vasculature that is perfectly balanced by pro- and anti-angiogenic molecules is composed of mature vessels with hierarchical distribution of arterioles, capillaries, and venules. Abnormal tumor vasculature typically lacks hierarchical structure and is composed of immature differentiated and undifferentiated vessels with increased permeability [1, 2]. The undifferentiated vessels frequently present with either collapsed or an absent lumen [3, 4]. Consequently, tumor vasculature is inefficient in carrying blood flow, resulting in a hypoxic tumor microenvironment, although the intra-tumoral microvessel density is commonly increased in contrast to the non-cancerous counterpart tissue.

The rationale of antiangiogenic therapy for solid tumors is founded on the fact that tumor growth and metastasis depend on the establishment of tumor vasculature, in which vascular endothelial growth factor (VEGF) signaling has been revealed to be one of the critical mechanisms [2, 5, 6]. Even while more anti-angiogenic agents become available in clinical practice, the survival benefit of cancer patients received from anti-angiogenic therapy remains limited [7–10], indicating the complexity of tumor angiogenesis and the necessity of targeting additional key components of tumor vasculature establishment [3, 11].

When the concept of tumor vasculature normalization was first introduced, it was believed that along with the pruning of the immature and undifferentiated vessels in a solid tumor, the remaining vasculature would also undergo shrinkage and be eventually unable to support tumor growth. Consequently, the tumor growth would be stalled and the tumor could be kept in dormancy [12, 13]. In an experimental treatment model of glioma using bevacizumab, an antibody targeting VEGF, the tumor vasculature could be normalized by low-dose bevacizumab treatment; however, no shrinkage was observed in the remaining vessels [14]. In contrast, the remaining vessels undergo remodeling and enlarge in size, supporting the continuous growth of the tumor [14]. This study clearly reveals two facts: first, tumor vasculature normalization itself cannot prevent solid tumor growth; second, after normalization, the remaining “normalized” vessels can undergo further remodeling and become more efficient in providing blood flow to support tumor growth.

In the present article, we discuss the origin of the remaining vessels after vasculature normalization treatment, as well as the alternative cellular origins of tumor vasculature, which are of great interest for improving the efficacy of antiangiogenic therapy.

### Co-opted vessels are prone to survival after tumor vasculature normalization

Vessels co-option, a procedure of hijacking the blood vessels in surrounding normal tissue along with the invasion of a solid tumor, has been recognized as an important approach to establish tumor vasculature, especially in more aggressive tumors [2, 15]. Vessel co-option may occur in tumors independently of sprouting angiogenesis, and sprouting angiogenesis is not always required for tumor growth.

The co-opted vessels are usually supported by pericytes from outside of the vessels. Pericytes are the supporting cells that stabilize blood vessels by accelerating the metabolism of lysophosphatidic acid [16, 17], while promoting endothelial cell survival via induction of autocrine VEGF-A signaling [18].

In human colorectal cancer metastasized to the liver, following antiangiogenic therapy with bevacizumab, the remaining resistant vessels are supported by pericytes and are much larger in diameter in comparison to capillary vessels, excluding the possibility of newly induced blood vessels by the tumor [19]. In mouse xenograft models of human ovarian and esophageal cancers, tumor vasculature normalization with bevacizumab treatment results in the increase of vessel pericyte coverage [20]. In a genetically engineered mouse model of pancreatic neuroendocrine tumors, long-term treatment with a vascular endothelial growth factor receptor-2 blocking antibody will generate refractory tumors. Inside the refractory tumors, the abundance of pericyte-covered co-opted vessels is increased [21]. In another experimental neuroblastoma model, persistent vessel co-option is the main mechanism for the tumor to evade antiangiogenic therapy [22].

Accumulating evidence suggests that the co-opted vessels can better survive after tumor vasculature normalization, and vessel co-option is an important approach of a solid tumor to evade antiangiogenic therapy [23].

### Vessel co-option accompanies vessel remodeling

Tumor-induced vessel remodeling provides better support for primary tumor growth by providing more blood flow [24]. During the natural expansion process of a solid tumor in the primary lesion [15] or in the metastatic lymph node [25], vessel remodeling always accompanies vessel co-option, as the co-opted vessel lumen enlarges prior to, and after co-option. The co-opted vessels undergo vessel remodeling in response to vasculature normalization, in observations dating to more than a decade past [22].

Important alterations during vessel remodeling to better support tumor growth include: (1) the tumor-induced extra-tumoral angiogenesis, more specifically, the generation of arterioles supported by multiple layers of pericytes, where the latter are believed to prevent vascular permeability and oxygen/nutrient exchange between circulation and normal tissue; (2) enlargement of the lumen space in the extra-tumoral vessels prior to vessel cooption; (3) following co-option indicated by the integration of the co-opted vessels and tumor vasculature along with the expansion of the tumor, the layers of pericytes become fewer, and eventually disappear, suggesting more feasibility of the oxygen/nutrient exchange between circulation and tumor tissue [15].

Vessel remodeling inside metastatic lymph nodes has been recognized in the high endothelial venules (HEVs) [25, 26]. HEVs belong to a special type of vessel only residing in lymphoid tissues, except the spleen. The normal function of HEVs is to maintain immune function by guiding the extravasation of naïve and central memory T cells from circulation to lymphoid tissue [27]. The key molecule for inducing lymphocytes extravassation is peripheral node addressin (PNAD) expressed by HEV endothelial cells. l-selectin is the homing receptor expressed on the cellular membrane of lymphocytes, which can recognize PNAD and induce localization and extravasation of the lymphocytes.

In the lymph node draining a solid tumor, the lack of conventional sprouting angiogenesis is accompanied by treatment resistance against bevacizumab [28]. Alternatively, dramatic remodeling of HEVs in the sentinel lymph node can be observed with larger lumen space, thinner wall, and more red blood cells inside the lumen compared with other non-sentinel lymph nodes, and all these changes can be induced by the primary tumor even before metastasis [25, 26]. After the arrival of metastatic cancer cells, the remodeled HEVs can be further co-opted into tumor vasculature along with the expansion of the tumor nests occupying the whole lymph node. The co-opted HEVs further undergo differentiation by losing their immunological marker PNAD, suggesting that following vessel co-option, the function of HEV has been switched from immune to carrying blood flow to better support the growing of metastatic tumor in the lymph node [25, 26]. In patients with squamous cell carcinoma of the tongue, increasing number of remodeled HEVs in the cervical lymph nodes associates with lymph node metastasis and poor patient survival [29]. Pioneering exploration on the underlying molecular mechanisms has revealed that bone morphogenetic protein-4 (BMP-4) is a negative regulator of HEV remodeling in the lymph node [30].

In summary, vessel co-option is commonly accompanied by vessel remodeling, which aims to provide more blood flow for tumor progression.

### Tumor cell-derived vasculature establishment

The capability of cancer cells to integrate into tumor endothelium, with or without the participation of endothelial cells to form a vessel-like network, has been observed for decades and termed as vasculogenic mimicry [31, 32]. Cancer stem cells are believed to be the culprit behind this phenomenon [33]. For example, glioblastoma stem-like cells have been found to be able to form tumor vasculature via endothelial differentiation [34]. Moreover, the cancer cell-originated vasculature has been suggested as an important mechanism underlying treatment resistance of anti-VEGF therapy [35]. For example, in an ovarian cancer mouse model, treatment of bevacizumab can inhibit tumor growth but accelerate metastasis with the formation of vasculogenic mimicry [36].

**Fig. 1:**
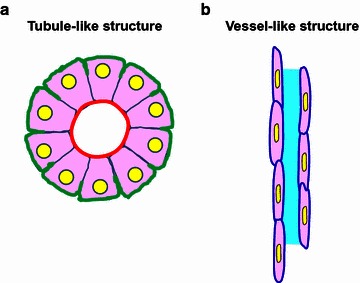
Illustration of the differences between a tumor cell-derived tubule-like structure and a vessel-like structure. **a** A cross section of a tubule-like structure showing multiple cuboidal cells forming a tubular structure. This kind of structure might be able to express different proteins in the apical membrane (*red*) and basolateral membrane (*green*). Polarization of nuclei (*yellow*) might be observed. **b** A longitudinal section of a vessel-like structure showing elongated cells with alternative staggered distribution of the nuclei, resulting in only one nucleus or no nucleus in any cross section. Notably, the *rod-like* structure of the nuclei indicates the trans-differentiation tendency from cancer cells to the cells forming blood vessel. Moreover, the vessel lumen (*light blue*) might be absent depending on different stages of development

The multipotentiality of cancer cells in transformation and trans-differentiation has been reported in numerous studies [37–39]. In addition to forming vessel-like structure, cancer cells with stem cell properties can also form branched tubular structure under certain circumstances. For example, T-47D breast cancer cells can form duct-like structure after the activation of c-MET receptor in vitro [40]. LA7 rat breast cancer cells with stem cell properties can form branched duct-like structure expressing luminal (K18), alveolar (β-casein) and myoepithelial (K14) markers [41]. Therefore, it is necessary to distinguish these two phenomena derived from cancer cells: vessel-like structure versus tubule-like (also known as duct-like) structure. Figure 1 illustrates the structural differences of these two morphological transformations of cancer cells.

We believe that the formation of vessel-like structures and tubule-like structures are two different directions of cancer cell trans-differentiation, and only vessel-like structures are related to the formation of tumor vasculature. The underlying molecular mechanisms of trans-differentiation from cancer cells to endothelial-like cells forming the vessel-like structure are of great interest, and there are ongoing studies focusing on this phenomenon.

The underlying molecular mechanisms of tumor-cell derived vasculature formation are far less illustrated. The αvβ5 integrin expressed in neuropilin 1-positive melanoma cells, which are highly aggressive cells, has been found to be responsible for vasculogenesis mimicry in melanoma [42]. This phenotype can be inhibited by cilengitide, a potent inhibitor of αν integrins activation [42]. As an epithelial-mesenchymal transition (EMT) regulator, zinc finger E-box binding homeobox 2 (ZEB2) can promote vasculogenic mimicry form by hepatocellular carcinoma cells [43].

Some other molecules, most of which are also expressed by cancer stem cells, have been found to be associated with vasculogenic tumor cells, including VE-cadherin, Nodal, hypoxia-inducible factor-1α, Roundabout-4, Sema4D, Ephrin-A1, Ephrin-B1, Ephrin-B2, EphA2, EphB2, EphB4, fibroblast growth factor 2, fibroblast growth factor receptor 1, CD133, Notch1, Nodal, Dll4, sonic hedgehog, Runx-1, ETV2, Mig-7, Twist-related protein 1, TIE1, uPA, TIE2, hepatocyte growth factor, matrix metalloproteinase 2 (MMP2), MMP9, and angiogenin [44, 45]. However, how these molecules orchestrate the phenotype of vasculogenic mimicry is mainly undetermined.

The close relationship between cancer stem cells and tumor cell-derived vasculature development suggests that targeting the stemness characteristics of cancer cells might be able to ultimately diminish the vasculature formation by cancer cell trans-differentiation.

### Vasculogenic mimicry and tumor metastasis

The close relationship between vasculogenic mimicry formation and metastasis has been repeatedly reported. For example, active caspase-3 simultaneously enhances the capacities of cellular motility and vascular mimicry formation of melanoma cells [46]. MicroRNA-124 (miR-124) represses both vasculogenic mimicry and motility of cervical cancer cells [47]. However, most of the studies could not validate that the cells contributing to vasculogenic mimicry are the same cells that metastasize to distant organs.

In a recent study, a causal relationship between vasculogenic mimicry and metastasis is proposed [48]. In this study using breast cancer animal models, the metastatic populations within a heterogenous tumor demonstrate their ability of forming vasculogenic mimicry to ensure other metastatic cells travel into circulation. The two identified proteins secreted by metastatic tumor cells, SERPINE2 and secretory leukocyte protease inhibitor (SLPI), are responsible for promoting vasculogenic mimicry. However, the tumor cells forming vascular network are morphologically altered to be thin layer cells similar to normal endothelial cells. There is no evidence to show that these cells could be transformed back to active metastatic tumor cells. Therefore, it is more reasonable to speculate that the metastatic cancer cell populations have a potential to form vasculogenic mimicry by sacrificing a small portion within the population and ensuring metastasis of the remaining cells; the cells forming vasculogenic mimicry are the ones most likely undergoing differentiation. This orchestrating action is partially supported by the evidence that in metastasis of pancreatic cancer cells, multiple clones of cancer cells occur in different phases of metastasis, indicating the heterotypic interactions between tumor subpopulations contributing to metastasis progression [49]. Our previous studies have also found that the low-metastatic cancer cells can be promoted to possess more aggressive behaviors by high-metastasis cancer cells via serglycin and interleukin-8 (IL-8) signaling [50–52].

In conclusion, establishing tumor vasculature is one of multiple events during the remodeling of tumor microenvironment for promoting tumor cell survival, growth, and spread. This attempt could be highly organized and coordinated among multipotent tumor cells and other normal host cells and host tissues. The well-known VEGF signaling pathway is crucial for sprouting angiogenesis from existing capillary endothelial cells as well as recruitment of circulating progenitor endothelial cells to tumor vasculature, which has become therapeutic targets in several cancer types. However, vessel co-option, remodeling of co-opted vessels, and forming vessel-like structure from tumor cells are some of the alternative approaches for a solid tumor to establish tumor vasculature as well as possible resistance against anti-VEGF therapy. The exploration of the underlying mechanisms of these alternative angiogenic approaches would not only widen our knowledge of tumor angiogenesis but could also provide novel therapeutic targets for better controlling cancer growth and metastasis.
